# Direct Detection of Lithium Exchange across the Solid Electrolyte Interphase by ^7^Li Chemical Exchange Saturation Transfer

**DOI:** 10.1021/jacs.2c02494

**Published:** 2022-05-30

**Authors:** David Columbus, Vaishali Arunachalam, Felix Glang, Liat Avram, Shira Haber, Arava Zohar, Moritz Zaiss, Michal Leskes

**Affiliations:** † Department of Molecular Chemistry and Materials Science, Weizmann Institute of Science, Rehovot 761000, Israel; ‡ Magnetic Resonance Center, Max-Planck Institute for Biological Cybernetics, Tübingen 72076, Germany; § Department of Chemical Research Support, Weizmann Institute of Science, Rehovot 761000, Israel; ∥ Institute of Neuroradiology, University Clinic Erlangen, Friedrich-Alexander Universität Erlangen-Nürnberg (FAU), Erlangen 91052, Germany

## Abstract

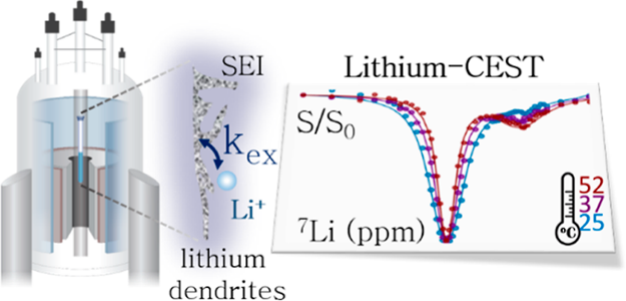

Lithium metal anodes offer a huge leap in the energy density of batteries, yet their implementation is limited by solid electrolyte interphase (SEI) formation and dendrite deposition. A key challenge in developing electrolytes leading to the SEI with beneficial properties is the lack of experimental approaches for directly probing the ionic permeability of the SEI. Here, we introduce lithium chemical exchange saturation transfer (Li-CEST) as an efficient nuclear magnetic resonance (NMR) approach for detecting the otherwise invisible process of Li exchange across the metal–SEI interface. In Li-CEST, the properties of the undetectable SEI are encoded in the NMR signal of the metal resonance through their exchange process. We benefit from the high surface area of lithium dendrites and are able, for the first time, to detect exchange across solid phases through CEST. Analytical Bloch-McConnell models allow us to compare the SEI permeability formed in different electrolytes, making the presented Li-CEST approach a powerful tool for designing electrolytes for metal-based batteries.

## 1 Introduction

Rechargeable batteries, in particular, lithium-based batteries, play a central role in the transition toward sustainable energy utilization. As such, there is global interest in developing battery cells and chemistries with improved performance and lifetime for large-scale applications such as electric transportation, grid-scale energy storage, and load leveling. One of the promising routes for achieving a leap in the cell performance is to replace the current graphite-based anodes with lithium metal, due to its high theoretical specific capacity (3860 mA h/g) and low negative redox potential (−3.04 vs standard hydrogen electrode), both result in ultrahigh energy density. However, the utilization of lithium metal is hampered by several fundamental challenges which include nonuniform lithium plating in the form of metallic dendrites and continuous reactivity with the electrolyte.^1,2^ The latter results in complex surface chemistry and the formation of the solid electrolyte interphase (SEI), a thin layer with a thickness of 10–100 nm, made of organic and inorganic phases.^3^ It is hard to overestimate the importance of the SEI and its central role in the cell’s performance: its strategic location at the anode–electrolyte interface makes its properties, namely, its composition and structure, a crucial factor determining lithium transport and deposition.^4,5^

Thus, the SEI, in general and in particular, on lithium metal has been the subject of numerous studies targeted at elucidating its chemical composition and morphology.^6−8^ However, the crucial effect of the SEI on the ion-transport process across the electrode–electrolyte interface is far less understood. Although experimental approaches are available for determining the phase composition of the SEI,^6^ insights into its permeability to lithium ions are much harder to obtain directly. Instead, information is mostly gained through electrochemical impedance spectroscopy (EIS) which requires extensive modeling for disentangling the multiple processes affecting interfacial resistance^9−12^ or through theoretical tools including density functional theory and ab initio molecular dynamics.^13−17^ Nuclear magnetic resonance (NMR) spectroscopy and mass spectrometry can be used to directly probe ion exchange across the electrode–electrolyte interface. To date, this has been achieved by lithium isotope exchange experiments which provide a global exchange rate that is a convolution of several processes, namely, ion desolvation, transport through the SEI, and exchange with the electrode surface.^18−24^ Another limitation of this method is that isotope exchange is difficult to use with cycled electrodes without perturbing the SEI layer formed on their surface. Another approach is the use of NMR exchange spectroscopy for determining the SEI–metal exchange rate.^25^ However, this requires the removal of all or part of the electrolyte which would otherwise dominate the spectra, and as such, it does not allow probing the SEI in its native form, which likely has significantly different transport properties in its dry form.^26^

Here, we employ a powerful NMR-based approach for directly probing ion exchange between lithium metal and the SEI in its native form. We utilize chemical exchange saturation transfer (CEST), a method which is commonly used in high-resolution ^1^H NMR and magnetic resonance imaging.^27−32^ Typically, CEST makes use of exchange between a large pool of ^1^H nuclei in the solvent (commonly H_2_O) and a small pool of exchangeable ^1^H on the molecule or biomolecule of interest. The spin population of the small pool (which is often invisible to direct NMR detection) is perturbed by a radio frequency (RF) pulse, and since this pool is in exchange with the large pool, part of this effect is transferred to the large pool population. As a result, the NMR signal of the large pool is reduced compared to its unperturbed signal, thereby allowing us to probe the small pool properties with higher sensitivity. This can be used to determine the identity and quantity of the chemical environment that is exchanging with the solvent, as well as the exchange rate between them.^33^

In this work, we show that the process of lithium exchange between the SEI and the metal can be efficiently captured with CEST, demonstrating the first implementation of CEST for detecting exchange between two solids. We first apply the approach to symmetric lithium battery cells, revealing the pronounced ^7^Li-CEST effect on dendritic lithium structures formed upon cycling. We then develop the approach systematically on dendritic lithium which is grown in situ in the NMR tube. The ability to detect entire dendritic structures with NMR (in contrast to bulk metal^34−37^) along with their high surface area offered by their fractal nature^1,38^ makes the exchange process between the metal dendrite and its SEI detectable with CEST. This is used to directly and efficiently compare the lithium permeability of the SEI formed in different electrolytes. Furthermore, modeling of the CEST profiles is used to quantify the metal–SEI exchange rate, and along with variable temperature measurements, we are able to determine the activation energy for lithium transport across the metal–SEI interface.

CEST offers a simple way to probe this otherwise invisible process which is fundamental for the performance of the battery cell. This is a powerful tool which can be employed for designing electrolyte systems with improved SEI properties. Moreover, the approach can possibly be extended to other emerging battery chemistries involving metal electrodes such as sodium, magnesium, and zinc.

## 2 Materials and Methods

### 2.1 Sample Preparation and Electrochemistry

Measurements on battery cells were performed on symmetric cells assembled in a PEEK casing^39^ using two lithium metal strips on top of copper mesh current collectors as electrodes, separated by a glass fiber separator (Whatman) soaked with 1M LiPF_6_ in a 1:1 mixture of ethylene carbonate, EC, and dimethyl carbonate, DMC (LP30, Solvionic electrolyte grade, <20 ppm water). For following dendrite growth, cells were cycled with current density in the range 1–3 mA/cm^2^ reversing the current direction every hour (see Figure S1 for the representative electrochemical plot).

Lithium dendrites were grown in an electrochemical setup designed to fit within a 5 mm NMR tube (see the Supporting Information). The electrochemical experiments were performed within an argon glovebox (O_2_, H_2_O < 0.5 ppm) where all materials were stored.

Two rectangular fresh lithium metal pieces (Sigma-Aldrich, 99.9% trace metal basis) were cut using scissors in dimensions of 3 mm × 5 mm × 0.38 mm and folded along their long axis onto Pt wires. The wires were connected to a Bio-Logic portable SP-50 potentiostat through sealed contacts at the back of the glovebox. Electrolyte solution (200 μL) was added to a standard 5mm NMR tube. Three types of electrolytes were used: (i) LP30, (ii) fluoroethylene carbonate, FEC, containing electrolyte, obtained by adding 10% (by volume) FEC (Gotion) to the LP30 solution, and (iii) 1 M lithium bis(trifluoromethanesulfonyl)imide, LiTFSI, in 1:1 v/v 1,2-dimethoxyethane/1,3-dioxolane, DOL/DME, which was made by dissolving LiTFSI salt (Sigma-Aldrich, dried under vacuum at 150 C for 48 h) in DOL and DME (Sigma Aldrich) which were thoroughly dried and stored with molecular sieves (3 Å).

The electrochemical device was inserted into the NMR tube, such that the Li pieces were fully immersed in the electrolyte, forming a symmetrical lithium cell. A constant current of 0.5 mA was applied to the cell for 4 h. After 4 h, dendrite formation was clearly visible in the form of gray material in the electrolyte. The electrochemical setup with the two metal pieces was removed from the NMR tube, and the gray fluffy dendrites were left suspended in the electrolyte. The tube was carefully sealed and taken to the NMR measurements.

### 2.2 NMR Experiments and Data Analysis

**Figure 1:**
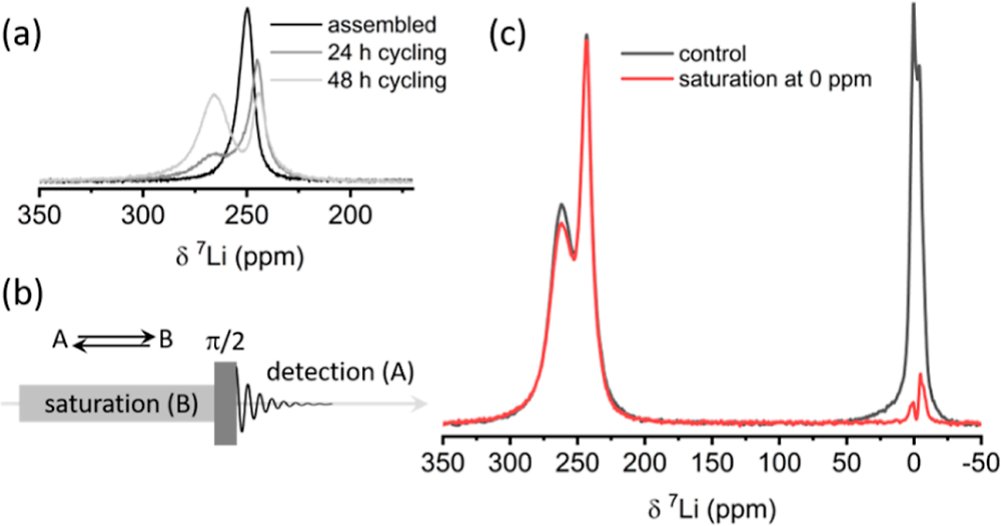
(a) ^7^Li static NMR spectra of the metal resonance from a symmetric battery cell before and after cycling with the LP30 electrolyte. (b) CEST pulse sequence with a soft saturation pulse applied on site B followed by an excitation on-resonance with site A. (c) ^7^Li spectrum of the symmetric battery cell after cycling (gray) and the spectrum acquired with a saturation pulse of 0.2 s and 500 Hz on the electrolyte resonance (red).

NMR measurements on symmetric battery cells were performed on a Bruker 9.4 T 400 MHz AVANCE III wide bore spectrometer with a static double resonance probe from NMR Service fit with a 1 cm solenoid coil. ^7^Li direct excitation experiments were performed with a 13 μs excitation pulse, and CEST experiments were performed using the sequence in Figure 1b with a 0.2 s-long saturation pulse at an RF amplitude of 500 Hz. The recycle delay in all experiments was set to 8 s.

NMR measurements on dendrite samples were performed on a 9.4 T, Bruker AVANCE III spectrometer with 400.35 and 155.6 MHz Larmor frequencies for ^1^H and ^7^Li, respectively, using a 5 mm BBI probe. The pulse duration was calibrated for each measurement separately (corresponding to a flip angle of 90° of about17 μs). Recycle delay was set to 8 s, allowing the probe to cool down from the saturation pulses. The longitudinal and transverse relaxation rates were determined for the lithium dendrites and the electrolyte using inversion-recovery and Carr–Purcell–Meiboom–Gill experiments, respectively. Quantitative measurements were performed by calibrating the excitation pulse and acquiring the lithium spectrum with a sufficiently long relaxation delay (compared to the electrolyte relaxation). CEST experiments were performed with the pulse sequence shown in Figure 1b. The saturation pulse duration and amplitude are specified in the different figure captions. In all experiments, the list of saturation frequencies contained a control experiment (acquired with saturation at +500 ppm from the lithium metal resonance) every 10 experiments to monitor the heating due to the RF pulses. This effect was then accounted for when plotting the CEST *Z*-spectra. Probe tuning was stable over the entire range of saturation frequencies.

The spectra were initially processed using Bruker Topspin software including phase and baseline corrections and peak integration. Further processing and analysis of the data were carried out in MATLAB (version 2018b).

## 3 Results and Discussion

### 3.1 ^7^Li CEST on Bulk Lithium Versus Dendrites

In order to test the feasibility and optimal setup for detecting lithium exchange across the metal–electrolyte interface, measurements were performed on symmetric lithium battery cells. Symmetric cells were assembled in a PEEK casing suitable for NMR measurements, as described in the Materials and Methods section. ^7^Li NMR spectra were acquired from the battery cell before cycling and following 24 and 48 h of cycling (Figure 1a). The spectrum acquired for the assembled battery cell displayed a single metal resonance centered at about 250 ppm. Following 24 h of cycling, a second resonance of a metallic lithium environment was observed at a slightly higher frequency, which grew significantly after 48 h of cycling. This environment corresponds to the formation of lithium dendrites in the battery, and the shift in its resonance frequency is associated with bulk magnetic susceptibility (BMS) effects and the different orientations of the metal strip and dendrites with respect to the external magnetic field.^37,40^

The full spectrum of the battery cell after cycling is shown in Figure 1c (gray). In the full spectrum, in addition to the metal resonances, the electrolyte environments are observed resonating around 0 ppm (the different resonances and variation in their position are again due to BMS effects for the electrolyte in different regions of the battery cell^41,42^). Upon contact of the metal electrodes with the electrolyte, spontaneous electrolyte reduction occurs, which is further exacerbated when fresh lithium is deposited during electrochemical cycling. These reduction processes lead to the gradual formation of the SEI.^2,11^ The SEI, which, in LP30, typically contains phases such as LiF, Li_2_O, Li_2_CO_3_, and various polymeric species all resonating around 0 ppm,^43−45^ cannot be sensitively detected in static NMR measurements in the presence of the dominating electrolyte resonance. This is due to the sharp and intense contribution of the electrolyte resonance, characteristic of mobile species in solution, in contrast to the ^7^Li resonance of the relatively nonmobile solid SEI environments. Resonances from the SEI span a broad range of frequencies due to anisotropic interactions (dipolar couplings and quadrupole broadening) and short transverse relaxation times. When the electrolyte was removed, the SEI resonances could indeed be detected (Figure S2). However, as the dry SEI will have significantly different permeabilities to lithium ions,^26^ we focus our investigation on the SEI in its native state, that is, immersed in the electrolyte.

For detecting Li exchange across the metal–electrolyte interface, we have employed the basic pulse scheme of the CEST experiment (Figure 1b). In order to observe exchange between two chemical environments A ⇌ B, we first apply a long soft (low amplitude) pulse aiming to saturate the resonances of environment B followed by detection of resonance A. If exchange is taking place at the time scale of the saturation and between sufficiently large populations in the two environments, it will result in partial saturation of resonance A and thus reduction in its signal. Results of these experiments on symmetric battery cells following cycling are shown in Figure 1c (red). When saturation was applied at 0 ppm, the electrolyte resonances were efficiently saturated. Interestingly, although no change was observed in the bulk metal resonance following saturation, the dendrites resonance was visibly reduced in intensity. This suggests that the exchange process, which can be detected with isotope exchange on metal strips,^22,23^ is not leading to sufficient saturation transfer between the two pools in the case of bulk lithium but can be detected through its effect on dendritic lithium. We speculate that this is due to the low surface area of the metal piece which limits the number of exchange events.

**Figure 2:**
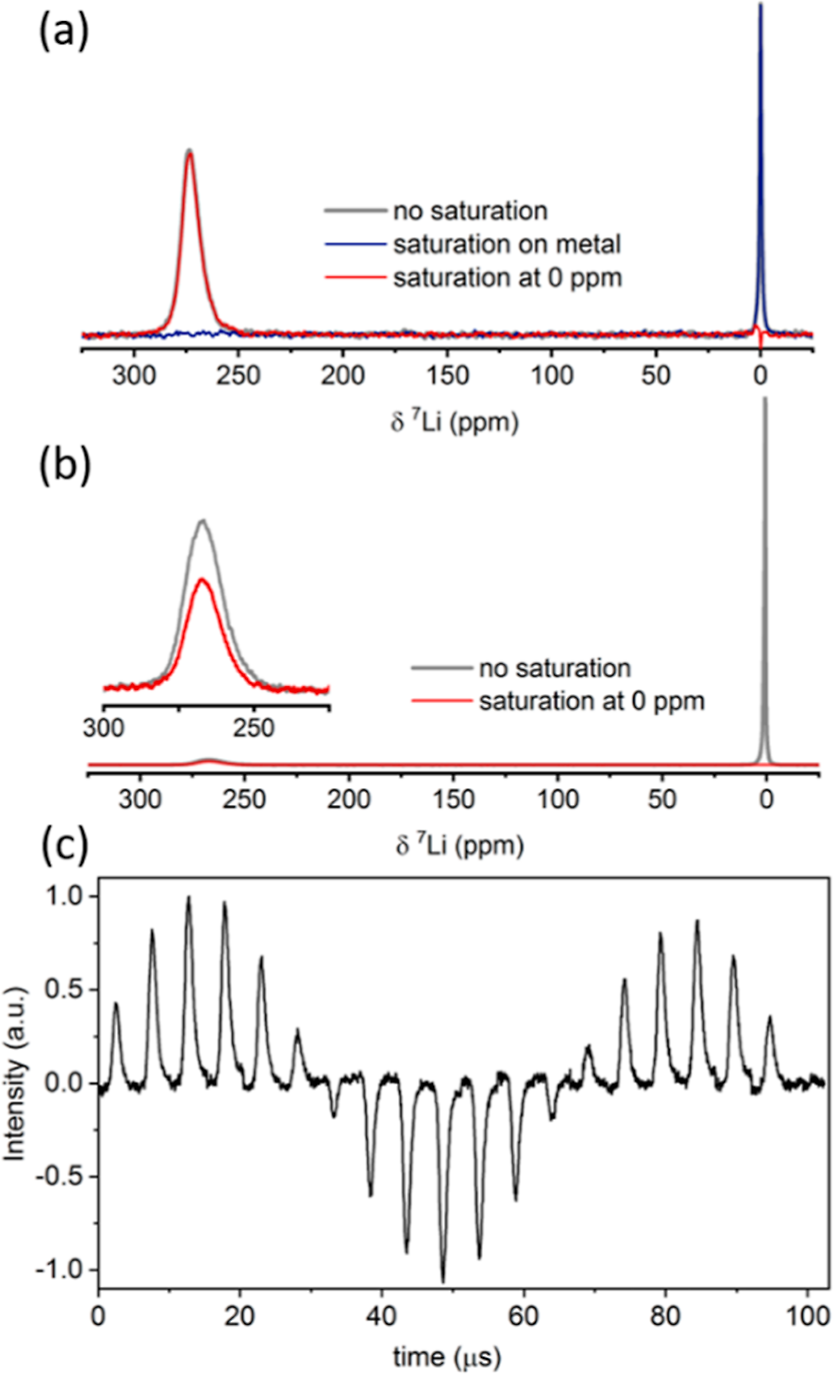
(a) ^7^Li spectrum of a Li metal piece immersed in LP30 (gray) and spectra acquired with a saturation pulse of 1 s and 500 Hz on the metal resonance (blue) and electrolyte resonance (red). (b) ^7^Li spectrum of dendrites immersed in LP30 (gray) compared with the spectrum acquired following a saturation pulse of 0.2 s and 800 Hz on the electrolyte. (c) ^7^Li nutation experiments performed on the dendrites.

The differences between bulk metal electrodes and dendritic lithium can be clearly appreciated in experiments performed separately on metal strips versus dendritic structures. CEST experiments performed on a lithium piece immersed in the electrolyte within the NMR tube do not show any effect on the metal resonance when the electrolyte resonance is fully saturated (Figure 2a). On the other hand, experiments performed on dendrites which were grown in the NMR tube (see the Materials and Methods section and Supporting Information) display a large CEST effect (Figure 2b). The difference is attributed to the dendrite specific surface area that is estimated to be 3 orders of magnitude higher than a piece of metal (see the Supporting Information for details). A ^7^Li nutation experiment (Figure 2c) revealed that the entire dendrite volume was efficiently excited and detected by the RF pulses, indicating that the thickness of the dendrites was lower than 1–2 μm (the RF penetration depth in lithium metal at 9.4 T).^34^ Due to the high surface to bulk ratio offered by the dendrites, application of the CEST sequence leads to a measurable decrease in the dendrite resonance following saturation at 0 ppm (where both the electrolyte and Li in the SEI resonate, Figure 1b). These experiments clearly show that dendrites enable detection of Li exchange across the metal interface with ionic lithium environments resonating at 0 ppm.

**Figure 3:**
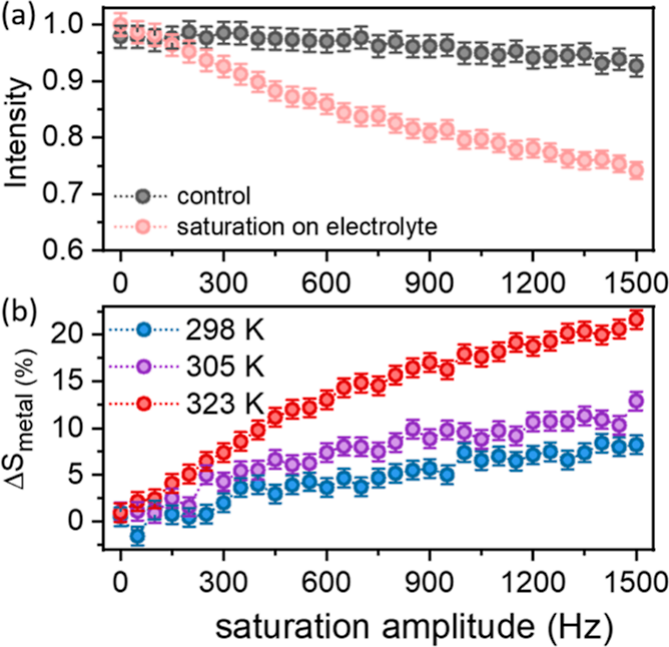
(a) ^7^Li integrated intensity of the dendrite resonance following 0.2 s saturation at +270 ppm from the metal signal (gray, control) and at −270 ppm (light red). Data were acquired at 323 K. (b) CEST effect quantified at different temperatures.

To confirm that the reduction in the metal signal is due to Li exchange, we performed two experiments. In the first, we varied the RF saturation amplitude B_1_ irradiating at 0 ppm (−270 ppm from metal) and compared the reduction in the metal signal to a control experiment where the irradiation was applied at higher frequency (+270 ppm from the metal). The results, plotted in Figure 3a, show that in the control experiment, the metal signal was reduced by up to 10% due to RF heating of the sample, while with saturation applied in the frequency range of the electrolyte/SEI resonances, the metal signal was decreased by 30%. Thus, quantification of the CEST effect (in %) should take into account RF heating as follows
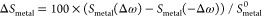
Here, Δ*S*_metal_ is the normalized change in the metal signal with saturation on the exchanging pool, resonating at Δω (offset with respect to the metal frequency Δω = ω_sat_ – ω_metal_), *S*_metal_(Δω), compared to reference saturation at −Δω. *S*_metal_^0^ corresponding to the metal signal without saturation.

The second confirmation that the observed effect is due to lithium exchange is the measured increase in the maximal CEST effect, from 7% to about 25%, observed with increasing the temperature from 298 to 323 K (Figure 3b). This increase, which is expected in the case of chemical exchange, rules out significant contribution from magnetization transfer (MT) through dipolar interactions. MT would have no or opposite dependence on temperature due to motional averaging of dipolar interactions.

### 3.2 Identifying the Exchanging Pool of Lithium Ions

**Figure 4:**
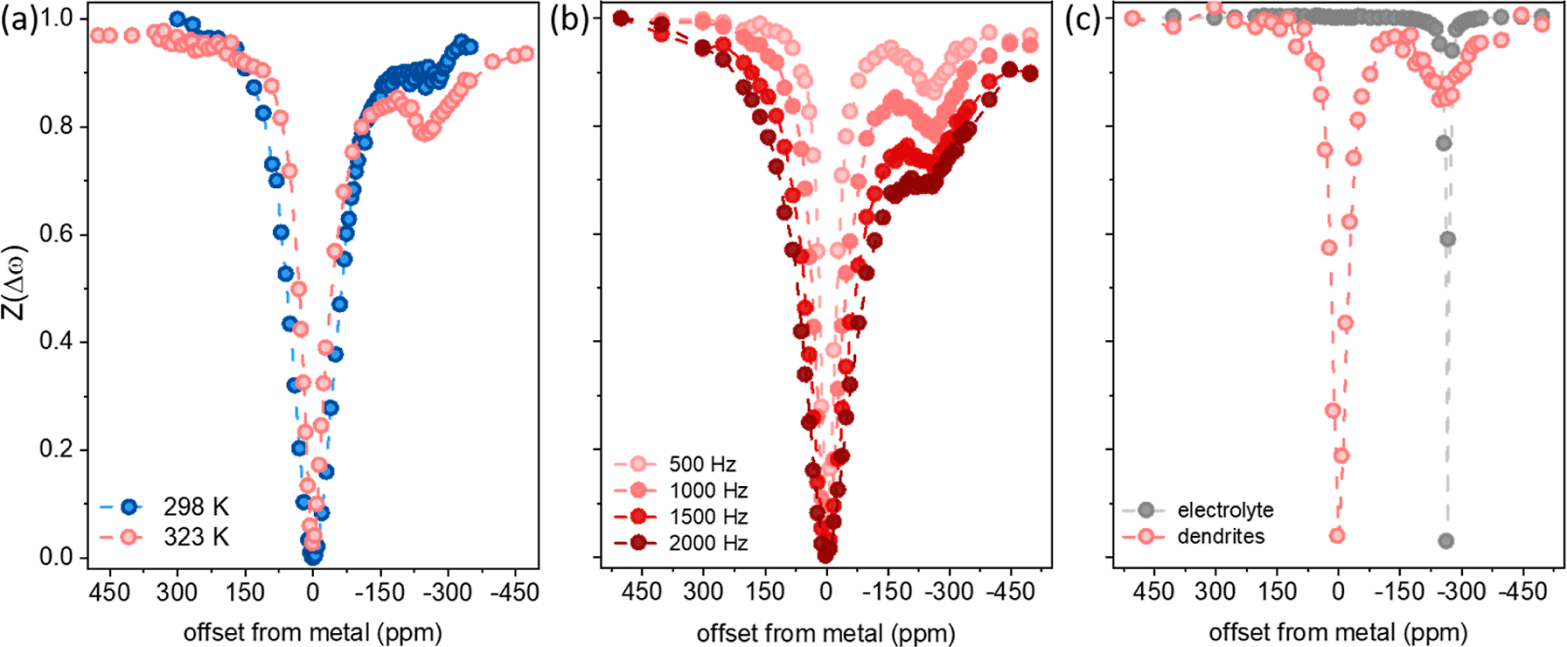
*Z*-spectra acquired from the dendrite signal in the LP30 electrolyte with 0.2 s saturation (a) at different temperatures with 500 Hz power, (b) at 323 K with varying saturation power, and (c) comparing the *Z*-spectrum of the dendrites and electrolyte at 323 K, 0.2 s saturation at 500 Hz.

For determining what lithium pool is exchanging with the lithium metal, the CEST experiment was performed as a function of the saturation frequency, Δω. Plotting the metal signal intensity as a function of saturation offset results in the *Z*-spectra plotted in Figure 4. Here, *Z*(Δω) was calculated using *Z*(Δω) = *S*_metal_(Δω)/*S*_metal_(Δω = 500 ppm) (RF heating during the experiment was further accounted for by control experiments as described in the Materials and Methods section). The *Z*-spectrum was acquired at 298 and 323 K (Figure 4a) and at 323 K at increasing saturation amplitude. In all spectra, we can see significant direct saturation effects of the Li dendrite signal corresponding to the reduction in signal intensity when the saturating pulse is around Δω = 0. The CEST effect corresponds to the reduction in metal signal when saturating at frequencies centered around 0 ppm (Δω = −270 ppm). As observed before, the magnitude of the effect increases with temperature (Figure 4a) and RF saturation amplitude (Figure 4b). Finally, we compare the *Z*-spectrum obtained when detecting the dendrite resonance at 270 ppm with that obtained when detecting the electrolyte resonance at 0 ppm (Figure 4c). This comparison reveals that the CEST effect is originating from exchange with a broad resonance spanning 50–100 ppm, extending beyond the range in which the electrolyte resonance is saturated. Furthermore, the electrolyte resonance was not affected by the saturation on the dendrite resonance. Finally, as observed in Figures 3b and 4b, the CEST effect on the dendrites was increasing with saturation amplitude, while the electrolyte signal could be easily saturated with low RF amplitudes (see Figure S7). These observations strongly suggest that the electrolyte does not contribute to the observed CEST effect and that the detected exchange process is between the dendrites and a broad diamagnetic Li resonance centered around 0 ppm (with width comparable to the SEI resonance observed in the ^7^Li spectrum measured from cycled and dried Li electrodes, Figure S2). The only Li pool that can lead to the observed CEST results is lithium ions stored in solid SEI layers which are undetectable by static NMR measurements in the presence of the electrolyte. Thus, the CEST experiment allows us to increase the sensitivity in detecting the SEI environments and provides a way to probe the exchange process between the SEI and the Li metal. Furthermore, these results highlight the advantage of employing CEST on a pure dendrite system which does not contain bulk lithium. This enables us to examine in detail the process of metal-SEI Li exchange and develop the ^7^Li-CEST methodology in a quantitative manner. This would be more challenging in a sample containing bulk lithium electrodes where the bulk Li and dendrite resonances can partially or fully overlap (see Figures 1c and S3). It is thus beneficial to remove the lithium electrodes as they reduce the overall CEST effect when they overlap with the dendrite resonance (see Figure S6).

### 3.3 Functionality of the SEI

As the CEST effect provides insights into the exchange between lithium dendrites and the SEI formed on them, it opens the way for evaluating the functionality of the SEI as an ion conductor. To this end, we compared the SEI formed in three different electrolytes using the ^7^Li CEST approach. In addition to LP30, dendrites were grown in LP30 with a 10% FEC additive (labeled LP30/FEC) and 1 M lithium bis(trifluoromethanesulfonyl)imide (LiTFSI) in a 1:1 mixture of dimethoxyethane (DME) and dioxalane (DOL) (labeled LiTFSI/DME/DOL). The same protocol (see the Materials and Methods section) was used to grow the dendrites in the three electrolytes, and they were then studied with ^7^Li CEST as described above. These electrolytes were chosen as they are often employed and evaluated based on their electrochemical performance and in lithium exchange experiments.^23,46−48^ As we will show, the ^7^Li CEST approach allows us to compare these electrolyte systems purely based on the effectiveness of their SEI as an ion conductor. This property cannot be simply extracted on its own from EIS or isotope exchange data. Both methods reflect, in addition to the metal–SEI transport, the effects of lithium desolvation and transport processes across the electrolyte–SEI interface, which would not affect our CEST measurements.

**Figure 5:**
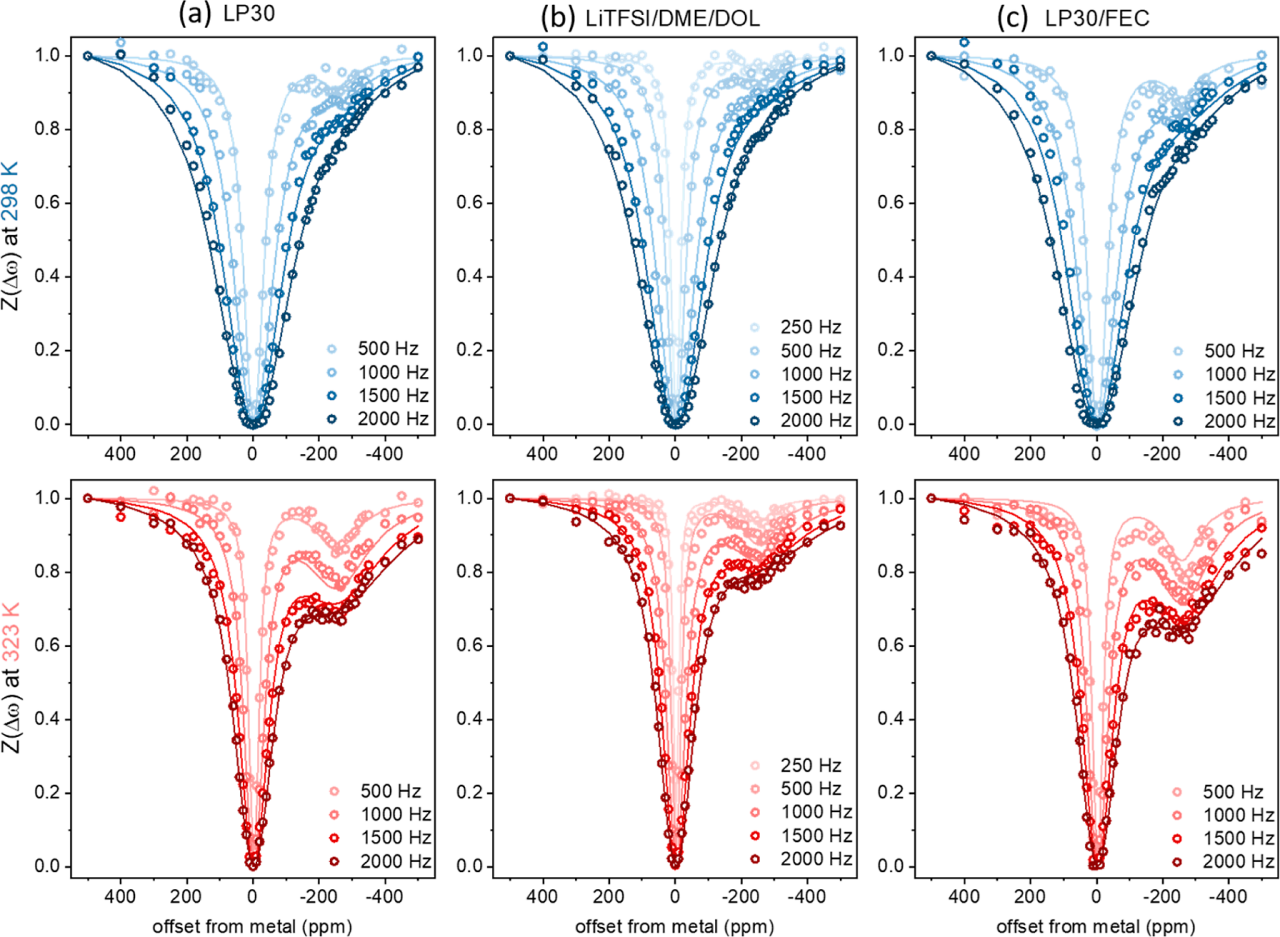
Experimental *Z*-spectra (circles) acquired from dendrites grown and immersed in (a) LP30, (b) LiTFSI/DME/DOL, and (c) LP30/FEC as a function of saturation amplitude with a 0.2 s saturation pulse at 298 and 323 K. Data were fitted (solid lines) by solving the two-pool BMC equation. Fit parameters are given in Table 2.

The experimental *Z*-spectra of the three electrolytes at 298 and 323 K at varying saturation amplitudes are shown in Figure 5a–c. Visual examination of the CEST profiles shows that, as observed for LP30, the effect increases with temperature. Qualitative comparison of the results suggests that adding FEC significantly increases the CEST effect (with a decrease in the metal signal by 40% compared to 30% without the additive), while the ether-based electrolytes lead to the lowest CEST effect.

#### 3.3.1 Quantification of the CEST Effect

In order to quantify the differences between the three systems, the Z-spectra were fitted using solutions to the two-pool Bloch-McConnell (BMC) differential equation.^49−51^ Here, the BMC equation describes the evolution of the magnetization under the effect of relaxation and RF saturation in the presence of an exchange process between the dendrite pool and the SEI pool with rates given by
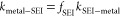
where *k*_metal–SEI_ and *k*_SEI–metal_ are the forward and backward exchange rates, respectively, and *f*_SEI_ corresponds to the ratio between the SEI and dendrite pool concentrations. Separating the exchange rate from the concentration of the exchanging pools requires simultaneous fitting of multiple B_1_*Z*-spectra.^52−56^

Comparison of the numerical solution^50^ with the analytical solution^51^ (see details in the Supporting Information) to the BMC equation on one set of data revealed that they provide similar results. Thus, the data were fitted with the computationally efficient analytical solution which was previously validated on other exchanging systems.^44−49^ This approach is valid under the assumption of a small SEI pool (fraction *f*_SEI_ ≪1) with negligible longitudinal relaxation compared to its transverse relaxation and exchange rates, *R*_1,SEI_ ≪ *R*_2,SEI_, *k*_SEI–metal_. Next, the results from the different electrolyte systems were fitted while using as input the multiple *Z*-spectra, acquired with varying saturation amplitude B_1,_ and the dendrite ^7^Li longitudinal relaxation rate. In general, the fits provided four output parameters that were optimized by simultaneous least-squares fitting of the *Z*-spectra at multiple B_1_: *k*_SEI–metal_, *f*_SEI_, and the transverse relaxation rates of the two pools *R*_2,SEI_ and *R*_2,dendrites_, with starting values and boundaries specified in Table 1. *R*_2,dendrites_ was also measured experimentally and was used to validate the results of the fits. First, we tested the results with all four fitting parameters free, which resulted in goodness of fit (GOF) values between 0.75 and 0.87 for the different systems and temperatures, with a relatively low GOF of 0.72 for the LP30/FEC system at 298 K (see the Supporting Information). To improve the fits and as the value of *f*_SEI_ did not vary much between data sets, its value was fixed to 0.02. This resulted in slight improvement in the GOF across all data sets. Additional fitting procedures were tested including expanding the number of exchanging pools to account for the heterogeneity of the SEI; however, these did not lead to significant differences in the results (see the Supporting Information for further details).

**Table 1:** Fitting Parameters and Boundaries

parameter	starting value	lower boundary	upper boundary
Δω_metal_ [ppm]	0	–10	10
*R*_2,metal_ [1/s]	2	0.2	4 × 10^4^
Δω_SEI_ [ppm]	–300	–400	–200
*f*_SEI_	0.0001	0.0000	9.0000
*k*_SEI–metal_ [Hz]	1000	1	10^6^
*R*_2,SEI_ [1/s]	50	0	5 × 10^4^

Despite some missing substructure, the rather simple fit model described above clearly tracks the experimental data, and especially its B_1_ dispersion, as can be seen in Figure 5a–c. The fitting parameters are summarized in Table 2. The simulations reproduce very well the direct saturation effect on the dendrites, as well as the decrease in the width of the effect with increasing temperature, due to the lower metal transverse relaxation rates *R*_2,dendrites_ (which match well the experimentally measured values). They also capture the breadth of the SEI resonance which is increasing with temperature, as reflected by the increase in transverse relaxation rate *R*_2,SEI_ (and fits rather well the breadth of the ^7^Li resonance from the dry SEI, Figure S2). Finally, the fits clearly show differences in the lithium *k*_SEI–metal_ rates measured in different systems.

**Table 2:** Experimental Relaxation Parameters and Fit Parameters Obtained from Two-Pool BMC Solution with Fixed *f*_SEI_ = 0.02^a^

	temperature (K)	*R*_1,metal_^exp^ (Hz)	*R*_2,metal_^exp^ (Hz)	*k*_SEI–metal_ (Hz)	*R*_2,metal_ (Hz)	*R*_2,SEI_ (kHz)	GOF (*R*^2^)
LP30	298	6.9	1750	64 ± 6	1395 ± 37	20 ± 9	0.85
	310	7.4	805	143 ± 8	709 ± 21	33 ± 8	0.86
	323	8	380	285 ± 13	393 ± 13	27 ± 8	0.86
LiTFSI/DME/DOL	298	6	1515	43 ± 5	1312 ± 29	30 ± 14	0.87
	323	7.8	360	141 ± 8	314 ± 11	38 ± 9	0.81
LP30/FEC	298	7	1670	91 ± 9	1456 ± 51	28 ± 13	0.75
	323	7.5	353	337 ± 18	397 ± 18	48 ± 8	0.74

a Uncertainties correspond to the 95% confidence interval of the nonlinear least-squares fitting procedure.

#### 3.3.2 Interpretation of CEST Profiles and Comparison of Different SEI Systems

We now turn to discuss what information can be gained from the CEST experiment and its analysis. Fitting the CEST profiles provides a direct measure of the SEI properties: this is reflected in the two parameters *k*_SEI–metal_ and *f*_SEI_ which together simply correspond to the efficiency of the SEI as an ion conductor. The higher these numbers are, the better the SEI should be in terms of its ability to conduct lithium ions between the electrode and the electrolyte. In principle, *f*_SEI_, which corresponds to the size of the SEI pool, compared with the dendrite pool, can inform us about the density of the SEI on the metal, that is, a higher fraction suggests denser SEI layers or higher coverage of the metal. As we used a simplified two pool model to represent the heterogeneous SEI, interpretation of *R*_2,SEI_ is limited since it corresponds to an average property of the different interphases. Nevertheless, its increase with temperature may be interpreted as increased exchange within the SEI phases, leading to overall higher contribution from the different interphases and thus broadening of the CEST effect.

**Figure 6:**
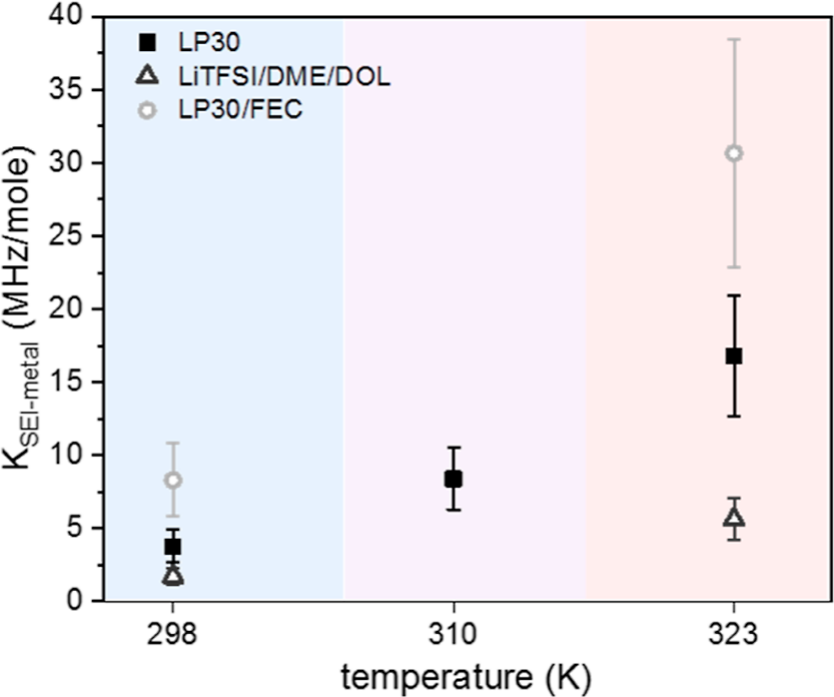
Exchange rates per mole of dendrites obtained from the two-pool BMC fit of the *Z*-spectra of dendrites grown in different electrolytes acquired with 0.2 s saturation at variable power at 298, 310, and 323 K (full *Z*-spectra and fits are shown in the Supporting Information).

In order to compare the different electrolytes through the fitted *k*_SEI–metal_, we have to take into account the amount of dendrites formed in each system. All samples were prepared with the same protocol, passing the same amount of current through the cell. However, the passed charge may distribute differently between the various processes occurring during galvanostatic cycling: smooth lithium plating, SEI growth, and dendrite formation. Therefore, to compare between different electrolyte systems, the exchange rates have to be normalized by the moles of dendrites collected in the NMR tube. Here, we make use of the quantitative nature of NMR and the ability to uniformly excite the dendrite resonance. Comparing the area of the dendrite resonance with the known amount of lithium contributing to the electrolyte resonance (taking into account longitudinal relaxation and the excitation bandwidth of the pulses), we are able to calculate the moles of dendrites formed (*n*_dendrites_), which is the highest in LiTFSI/DME/DOL, followed by LP30 and finally LP30/FEC (see the Supporting Information). Using this result, we are able to obtain a comparable measurement of the permeability of the SEI through *K*_SEI–metal_ = *k*_SEI–metal_/*n*_dendrites_ which is shown in Figure 6. We note that in this comparison, we assumed that the entire dendrite pool is taking part in the exchange process, meaning that we neglect any effects that may arise due to different dendrite morphologies (further discussion about this assumption and the exchange active surface area of the dendrites can be found in the Supporting Information). These results clearly show that the SEI formed with the FEC additive is much more permeable than that formed on LP30, with the ether system leading to the slowest exchange rates across the metal–SEI interface. This fits well the improved performance typically reported for Li metal cycled with FEC containing electrolytes.^23,48,57,58^ This is often attributed to a more compact and multilayered SEI with increased formation of LiF domains at the SEI–metal interface.^46,47,59^

Finally, the ability to determine the exchange rate at different temperatures allows us to calculate the activation energy for Li transport from the SEI to the metal, using the Arrhenius equation. Here, we performed this process for LP30 and extracted an activation barrier of 47 ± 2 kJ/mole corresponding to 0.47 ± 0.02 eV for Li transport. This value falls within the predicted calculated range for lithium migration in various inorganic SEI components,^15^ in particular, Li_2_CO_3_, which is likely a major component formed with the LP30 electrolyte. We believe that this is the first direct determination of the energy barrier for the SEI in its native form. Previous exchange experiments performed on the partially dried SEI yielded similar values for ether-based electrolytes (0.16 ± 0.07).^25^ Our exchange rate measurements for the ether-based system at two temperatures also suggest a slightly lower activation barrier in this electrolyte compared to LP30; however, additional temperature measurements would be required to determine an accurate value. We note that the energy barrier for exchange is the most relevant property when comparing lithium transport across the SEI under cycling, while the value of the exchange rate will change under electrochemical conditions.

## 4 Conclusions

Lithium CEST was introduced for the first time as a means to measure ionic exchange across solid–solid interfaces. The approach is straightforward to apply, requires a minimal experiment time (compared to 2D exchange spectroscopy), and provides a simple way to qualitatively compare different SEI and electrolyte systems. Established numerical and analytical Bloch-McConnell models can be extended to Li CEST and used to quantitatively determine the exchange parameters of the system.

The implementation of the approach to lithium dendrites provided a highly reproducible setup, which allowed us to carefully examine the factors affecting the CEST measurements and their quantitative analysis. This setup enabled comparison of the SEI permeability of three common electrolyte systems with clear superiority observed when FEC is used as an additive. Thus, CEST measurements provided evidence that the often-reported improvement in electrochemical performance observed with FEC is due to its positive SEI properties. As such, Li-CEST emerges as an efficient approach to design new electrolytes which would give rise to beneficial SEI layers.

Our preliminary investigations suggest that the CEST approach can also be employed in Li–metal battery cells, providing qualitative comparison between systems, which can possibly be made quantitative provided sufficient spectral separation is obtained between the bulk and dendritic lithium resonances. Finally, we expect that this approach can be easily extended to probe ion exchange in other metal electrodes such as sodium and, depending on the available sensitivity, also magnesium and zinc.

## Supporting Information Available

Photographs of the experimental setup and calculation of the lithium dendrite dimensions, additional experimental data, representative electrochemical profile for the in-situ NMR battery cell and dendrite formation in the NMR tube, ^7^Li spectra from battery cells and washed Li electrodes, ^7^Li-CEST measurements performed on dendrites with and without the lithium electrode, electrolyte saturation at variable saturation amplitudes, quantification of the dendrites formed and variable temperature *Z*-spectra of dendrites in LP30, additional information on *Z*-spectra fitting, fitted BMC equation, fitting results under different assumptions, and calculation of the energy barrier for lithium migration (PDF)
