# Supplementary material for: Direct Detection of Lithium Exchange across the Solid Electrolyte Interphase by 7Li Chemical Exchange Saturation Transfer
Source: J Am Chem Soc. 2022 May 30;144(22):9836–44. doi: 10.1021/jacs.2c02494 (PMC9185740; doi:10.1021/jacs.2c02494)
Supplement: Supplementary file 1 — ja2c02494_si_001.pdf [file ja2c02494_si_001.pdf]

Supporting Information

Direct Detection of Lithium Exchange across the Solid Electrolyte Interphase by ^{7}Li Chemical Exchange Saturation Transfer

David Columbus[(1)], Vaishali Arunachalam[(1)], Felix Glang[(2)], Liat Avram[(3)], Shira Haber[(1)], Arava Zohar[(1)], Moritz Zaiss[(2,4)], Michal Leskes [(1)]*

(1) Department of Molecular Chemistry and Materials Science, Weizmann Institute of Science, Rehovot, 761000, Israel
(2) Magnetic Resonance Center, Max-Planck Institute for Biological Cybernetics, Tübingen, 72076, Germany
(3) Department of Chemical Research Support, Weizmann Institute of Science, Rehovot, 761000, Israel
(4) Institute of Neuroradiology, University Clinic Erlangen, Friedrich-Alexander Universität Erlangen-Nürnberg (FAU), Erlangen, 91052, Germany

*michal.leskes@weizmann.ac.il

1. Experiments on symmetric battery cells

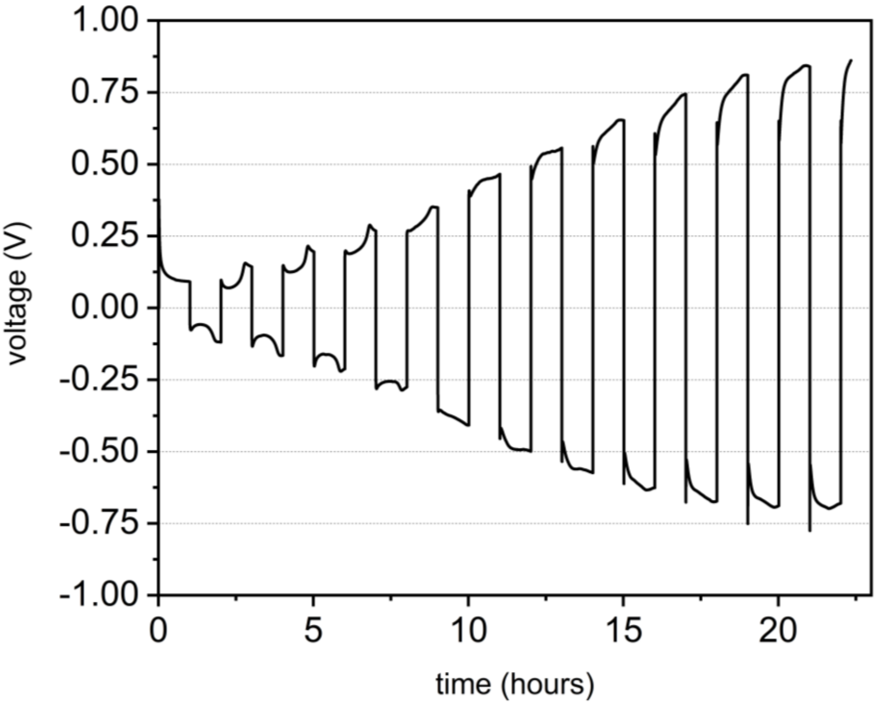

Figure S1 Voltage profile of a symmetric lithium battery cell cycled in LP30 electrolyte for 24 hours. The current density used was 3 mA/cm^2 and the current direction was reversed every hour.

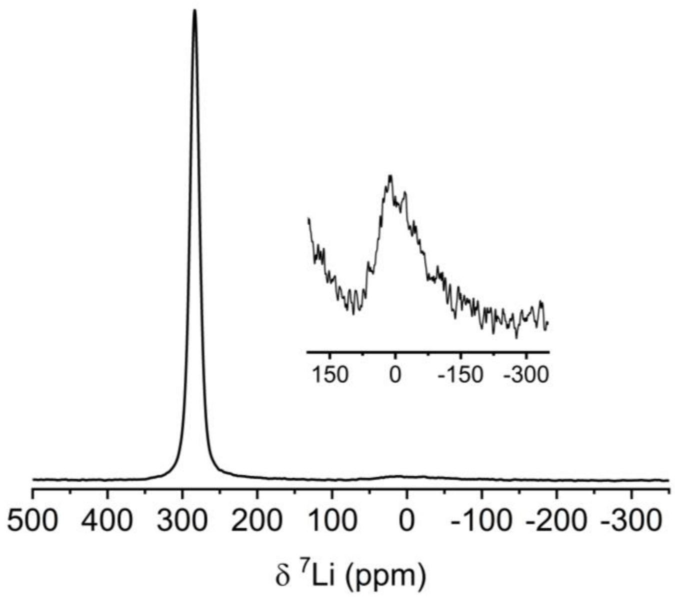

Figure S2 ^{7}Li static NMR spectrum of an Li electrode extracted from a battery cell following cycling, rinsed thoroughly with DMC, dried overnight under vacuum and packed in the NMR rotor. Inset shows the spectral region of the SEI.

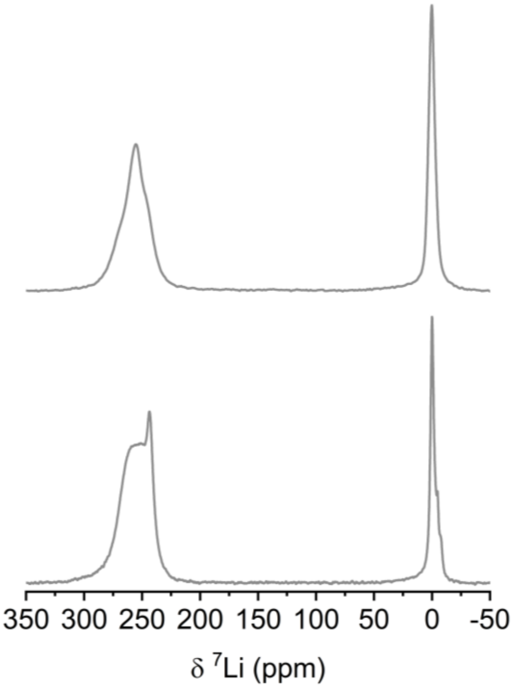

Figure S3 Representative ^{7}Li static spectra of symmetric Li cells showing the overlap between dendrites and bulk Li resonance.

2. Dendrites dimensions and exchange-active fraction

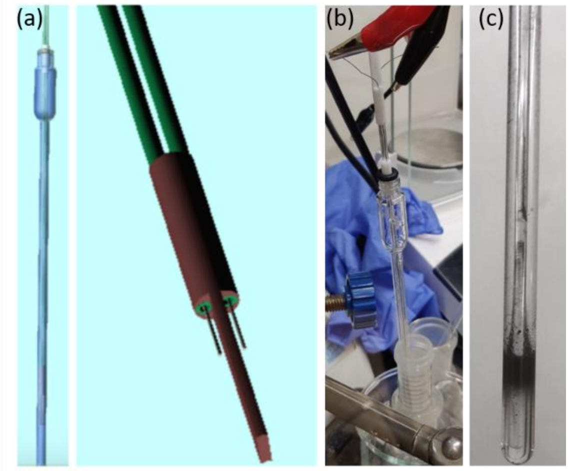

Figure S4 (a) Schematic design of the electrochemical cell with two Pt wires. Lithium electrodes were pressed on top of the Pt wires. (b) A photo of the cell inside the NMR tube, connected to the potentiostat inside the glove box. (c) A photo of the dendrites collected in the NMR tube (with sealed capillary containing deuterated solvent as NMR lock signal).

Dendrites were grown as described in the main text, using a custom-made electrochemical cell that can fit within an NMR tube (Figure S4). ^{7}Li nutation experiments confirmed that all residual metallic lithium in the NMR tube corresponds to dendritic lithium.

To extract the numerical values of the physical parameters of interest, an estimation of the surface area (SA) of the dendrites is required. The CEST effect (i.e., the fraction of the total signal that is decreased due to exchange interaction with a saturated pool) is correlated with the number of exchange events occurring during the saturation pulse. The number of events can be written such that:

$$\#\ of\ exchange\ events \propto k_{ex} \cdot SA$$

Meaning, the exchange rate and the surface area are inversely proportional to one another in terms of the CEST effect, such that any uncertainty in the estimation of the SA directly affects the confidence in the value of k_{ex}. To estimate the SA of dendrites, we used a method that involves NMR relaxation measurements of the solvent protons.[1] Solvent ^{1}H T_1 and T_2 relaxation values were measured (inversion recovery for T_1; CPMG for T_2) on four different samples: (i) LP30 electrolyte; (ii) LP30 + 1 Li metal piece; (iii) LP30 + 3 Li metal pieces; (iv) LP30 + Li dendrites. The lithium dendrites were formed by an electrochemical process where a constant current was applied in a symmetrical Li metal cell. The Li metal pieces were cut

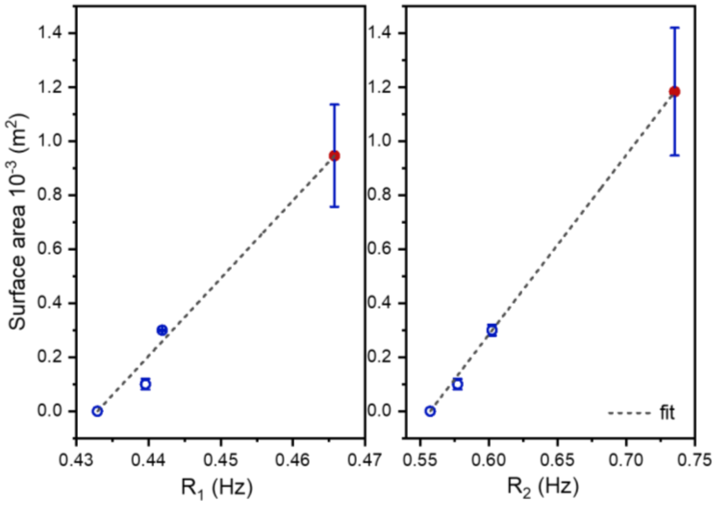

Figure S5 ^{1}H (a) R_1 and (b) R_2 relaxation measurements of the solvents in LP30 with metal pieces (blue circles) and with dendrites (red circle).

by an oval punch to produce fixed size pieces with weight of 10 mg. In samples (ii)-(iv), the same amount of electrolyte was added, and the samples were kept in the gloves-box for ~24h to let the SEI formation finish and stabilize.

According to Equation (3) in ref. [1] , the SA of the solid is linear with the relaxation rate, so a line can be fitted using three data points of samples (i)-(iii) (where both relaxations and SA values are known), and the SA of sample (iv) was estimated by extrapolation. The equation that was used to fit was simply $SA = a \cdot R_{1,2} + b$. The results from the experiment are shown in Figure S5. It can be seen from the plots that the SA of the dendrites is ~10-times higher than the SA of 1 Li metal piece (by taking the mean value of the analysis of the two relaxation processes).

To find the specific SA (i.e., SA per mole of Li), the SA values were divided by the number of Li moles in the sample. The number of Li moles in a metal piece:

$$n_{Li-piece} = \frac{mass}{MW} = \frac{10 \cdot 10^{-3} g}{6.941 \frac{g}{mol}} = 1.4 \cdot 10^{-3} mol$$

The number of Li moles in the dendrites is calculated by taking the electrochemical parameters that were used to form the dendrites. This amount was scaled by a factor of 3 according to quantitative NMR measurements (where the dendrites signal was quantified based on the known amount of Li in the electrolyte) which showed that about 25-40% of the charge passed resulted in dendrite formation (see also Figure S8).

$$n_{dendrites} = 0.33 \frac{I \cdot time}{F} = 0.33 \frac{0.5 \cdot 10^{-3} \frac{C}{sec} \cdot 14{,}400 sec}{96485 \frac{C}{mol}} = 2.5 \cdot 10^{-5} mol$$

Thus, the specific SA are given by:

$$SA_{Li-metal} = \frac{measured\ SA}{n_{Li-piece}} = \frac{1 \cdot 10^{-4} m^2}{1.4 \cdot 10^{-3} mol} \cong 0.07 \frac{m^2}{mol}$$

$$SA_{dendrites} = \frac{extrapolated\ SA}{n_{dendrites}} = \frac{1 \cdot 10^{-3} m^2}{2.5 \cdot 10^{-5} mol} \cong 40 \frac{m^2}{mol}$$

We can see that dendrites have specific SA that is 600 times higher than Li metal. This is in rough agreement with the results of previous research[2] where it was concluded that SA of a cycled Li metal anode is about 150 times higher than the geometric SA.

Next we would like to consider what fraction of the dendrites is participating in the exchange process with the SEI, namely what fraction of the detected dendrite signal is contributing to the CEST effect. To address this question, we first estimate the moles of Li that cover a given

area. We can provide a very rough (yet rationalized) estimation by simply using Li metal density. From the value of the density we can extract values of 1D and 2D Li metal densities. Assuming a perfectly symmetric Li metal cube with an edge length of 1 cm and volume of 1 ml, the number of Li atoms per volume is given by:

$$\frac{density}{MW} \cdot N_A = \frac{0.534\,\frac{g}{cm^3}}{6.941\,\frac{g}{mol}} \cdot 6.022 \cdot 10^{23}\,\frac{atoms}{mol} = 4.63 \cdot 10^{22}\,\frac{atoms}{cm^3}$$

Thus, the 1D density is given by:

$$\frac{atoms}{m} = \sqrt[3]{4.63 \cdot 10^{22}\,\frac{atoms}{cm^3}} \cdot 100\,\frac{cm}{m} = 3.6 \cdot 10^{9}\,\frac{atoms}{m}$$

And the 2D density specified in moles per area:

$$density_{2D} = \frac{(3.6 \cdot 10^{9}\,\frac{atoms}{m})^2}{N_A} = \frac{1.29 \cdot 10^{19}\,\frac{atoms}{m^2}}{6.022 \cdot 10^{23}\,\frac{atoms}{mol}} = 2.15 \cdot 10^{-5}\,\frac{mol}{m^2}$$

From this we can estimate the moles of Li at the surface of a metal piece:

$$n_{surface}^{Li\,piece} = density_{2D} \cdot SA = 2.15 \cdot 10^{-5}\,\frac{mol}{m^2} \cdot 1 \cdot 10^{-4} m^2 = 2.15 \cdot 10^{-9} mol$$

To estimate the moles of Li at the surface of the dendrites:

$$n_{surface}^{dendrites} = density_{2D} \cdot SA = 2.15 \cdot 10^{-5}\,\frac{mol}{m^2} \cdot 1 \cdot 10^{-3} m^2 = 2.15 \cdot 10^{-8} mol$$

Finally, we can determine the fraction of Li atoms on the surface of the metal piece and the dendrites:

$$f_{Li\,piece\,surface} = \frac{n_{surface}^{Li\,piece}}{n_{Li\,piece}} = \frac{2.15 \cdot 10^{-9} mol}{1.4 \cdot 10^{-3} mol} \approx \mathbf{{}^{1}/_{650000}}$$

$$f_{Li\,dendrites\,surface} = \frac{n_{surface}^{dendrites}}{n_{dendrites}} = \frac{2.15 \cdot 10^{-8} mol}{2.5 \cdot 10^{-5} mol} \approx \mathbf{{}^{1}/_{1200}}$$

Theoretically, it can be assumed that the fraction of Li atoms on the dendrites surface is proportional to the fraction of the exchangeable site, which in turn dictates the observable CEST effect. However, experimentally, we get that the reduction of the signal in the CEST experiment can reach up to 30% and even more, meaning that the *actual* exchangeable fraction is *at least 0.3 = 1 / 3.33 >> 1 / 1200*. This difference can be accounted for by a significant underestimation of the SA of the dendrites. Such an underestimation may originate from an underestimation of the actual SA of the Li metal piece, which was taken as the geometric SA in the calculation. Any deviation from this will linearly affect the value of SA for the dendrites. Furthermore, it is possible that within the dendrites there is very fast self-diffusion of Li atoms,

faster than in the bulk metal, due to increased number of structural defects. If self-diffusion within the dendrites is much faster than the saturation time scale (200 ms) we can expect this can result in non-surface Li atoms actively contributing to the CEST.

In order to emphasize the difference in the CEST effect between dendrites and a metal piece, we measured the CEST effect, ΔS_{metal} as defined in the main text, as a function of the saturation amplitude on a sample of Li dendrites together with the Li metal electrodes (cycled and measured in LiTFSI/DME/DOL electrolyte). Figure S6 shows a comparison between the two samples, with and without Li metal pieces: We can clearly see that adding bulk Li pieces decreased the CEST effect significantly. The reason for this is that out of the total signal of the Li pieces, only a tiny fraction of atoms (mostly in the high surface area dendrites) is actually taking part in the exchange process. Thus, a fixed fraction of redundant Li-metal signal does not contribute to the measured CEST effect at all. These findings clearly show that Li-dendrites provide an excellent system for CEST measurements.

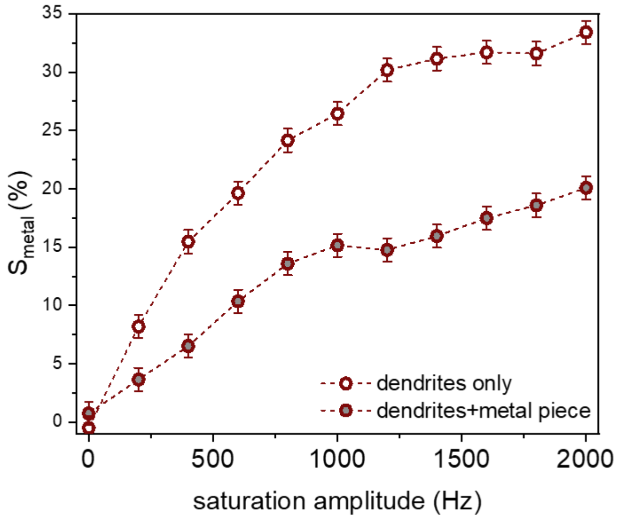

Figure S6 The CEST effect measured with dendrites grown in LiTFSI/DME/DOL, measured at 323 K with 0.2 sec saturation at -270 ppm and 800 Hz with and without the lithium electrode.

3. Electrolyte saturation

The electrolyte saturation vs. saturation power is shown in Figure S7 for LP30. This data shows that the electrolyte resonance is easily saturated with an RF amplitude of 500 Hz.

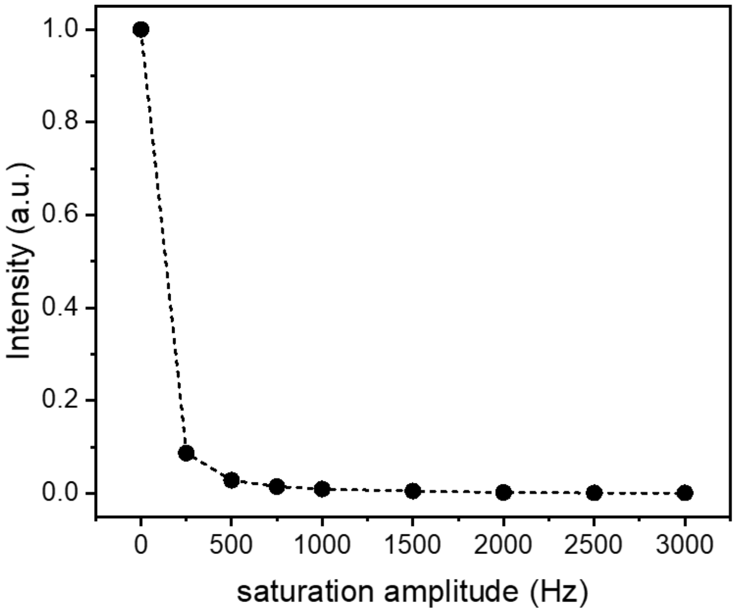

Figure S7 The intensity of ^{7}Li LP30 resonance measured after 0.2 sec saturation at varying power level.

4. Quantification of dendrites formation

The amount of dendrites formed in the different electrolytes in 4 hours of constant current of 0.5mA was quantified based on the integrated area of the ^{7}Li dendrites resonance. The integrated area was then compared with the integrated area of the electrolyte resonance (measured under steady state and corresponding to 200 μmole based on 200μl of 1M Li salt)

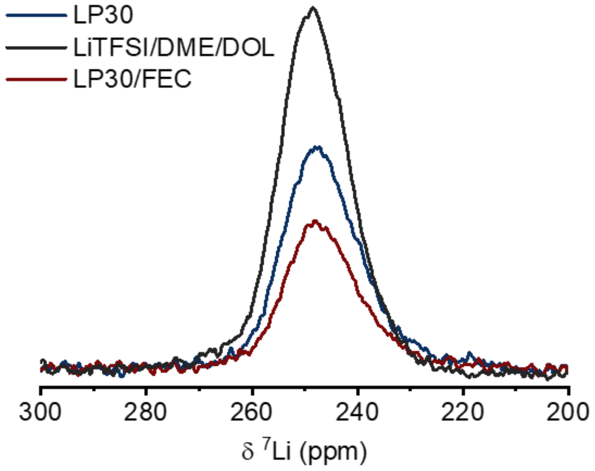

Figure S8 ^{7}Li spectra showing the lithium dendrites resonance grown in different electrolytes. Spectra were collected at steady state conditions.

	peak area	μmole
**LP30**	$1.23 \cdot 10^{8}$	17 ± 3
**LiTFSI/DME/DOL**	$1.81 \cdot 10^{8}$	25 ± 5
**LP30/FEC**	$7.75 \cdot 10^{7}$	11 ± 2

Table S1 Quantification of dendrite formation based on NMR.

to determine the moles of dendrites collected in the NMR tube. The dendrites resonance and the corresponding moles formed are shown in Figure S8 and Table S1, respectively.

5. Z-spectra fitting

The Z-spectra of the three electrolyte systems were fitted using the analytical solution to the two pools Bloch-McConnell (BMC) equations describing six coupled first order linear differential equations:[3–5]

$$\frac{d\vec{M}}{dt} = \boldsymbol{A}\vec{M} + \vec{C}$$

With the magnetization vector:

$$\vec{M} = (M_{d,x,}M_{d,y,}M_{d,z,}M_{s,x,}M_{s,y,}M_{s,z})^T$$

With d and s stands for dendrites and SEI pools, respectively, and:

$$\boldsymbol{A} = \begin{bmatrix} \boldsymbol{L}_d - f_s\mathbf{K} & \mathbf{K} \\ f_s\mathbf{K} & L_s - \mathbf{K} \end{bmatrix}$$

$$\boldsymbol{L}_i = \begin{pmatrix} -R_{2i} & -\Delta\omega_i & 0 \\ \Delta\omega_i & -R_{2i} & \omega_1 \\ 0 & -\omega_1 & -R_{1i} \end{pmatrix};\ \mathbf{K} = \begin{pmatrix} k_s & 0 & 0 \\ 0 & k_s & 0 \\ 0 & 0 & k_s \end{pmatrix};\ \vec{C} = (0,0,R_{1d}M_{d,0,}0,0,R_{1s}M_{s,0})^T$$

With i=d,s, $\omega_1 = \gamma B_1$where γ is the nuclear gyromagnetic ratio and B_1 the saturation amplitude, $\Delta\omega_i$ the saturation frequency $k_s = k_{SEI-metal}$.

First the equation was fitted with all four fitting parameters free. The results of this fit are given in Table S2. To reduce the error, we have also tested fitting the data while fixing the fraction of the SEI in comparison to the dendrite pool (Table 1 in the main text). Finally, the value of the SEI transverse relaxation was fixed to 40kHz with all other parameters free (Table S3). As expected, fixing one parameter leads to improvement in the goodness of fit (GOF) and as f_{SEI} is relatively constant, we used this setup for the main text.

As discussed in the main text, it is known that the SEI is heterogeneous, and is made of several phases and thus it is not clear if the single-CEST-pool assumption is valid, although the single pool model fits the data surprisingly well. To test this, a 3-pool model with a second exchanging

	Temperature (K)	$k_{SEI-metal}$ (Hz)	f_{SEI}	$R_{2,metal}$ (Hz)	$R_{2,SEI}$ (kHz)	GOF (R^2)
**LP30**	298	82 ± 76	0.016 ± 0.014	1396 ± 40	24 ± 24	0.83
	310	157 ± 44	0.019 ± 0.004	708 ± 22	36 ± 10	0.85
	323	412 ± 60	0.015 ± 0.002	399 ± 13	60 ± 8	0.87
**LiTFSI/DME/DOL**	298	53 ± 34	0.017 ± 0.009	1320 ± 30	26 ± 19	0.86
	323	181 ± 46	0.016 ± 0.003	319 ± 12	41 ± 11	0.77
**LP30/FEC**	298	6 ± 419	0.284 ± 20	1454 ± 59	3 ± 183	0.72
	298	66 ± 6	0.02*	1456 ± 50	23 ± 11	0.75
	323	228 ± 42	0.027 ± 0.004	395 ± 17	43 ± 8	0.76

Table S2 Fit parameters obtained from two-pool BMC solution with all parameters free. Asterisk indicates that in this fit f_{SEI}=0.02.

pool was tested, which did not lead to a big improvement in the GOF and at the same time only added a broad contribution centred at -150 or -350 ppm (data not shown). Thus the simpler 2-pool model was favoured following Occam's razor. However, if the general $R_{2,SEI}$ is much larger than the width of a possible inhomogeneous peak dispersion, a single pool assumption might still be a reasonable approximation.

	Temperature (K)	$k_{SEI-metal}$ (Hz)	f_{SEI}	$R_{2,metal}$ (Hz)	GOF (R^2)
**LP30**	298	99 ± 42	0.014 ± 0.005	1388 ± 37	0.85
	310	165 ± 35	0.018 ± 0.003	706 ± 20	0.86
	323	350 ± 52	0.017 ± 0.002	409 ± 13	0.86
**LiTFSI/DME/DOL**	298	51 ± 21	0.017 ± 0.007	1310 ± 29	0.86
	323	179 ± 38	0.016 ± 0.003	315 ± 10	0.81
**LP30/FEC**	298	67 ± 27	0.027 ± 0.01	1448 ± 50	0.74
	323	222 ± 37	0.028 ± 0.004	395 ± 16	0.75

Table S3 Fit parameters obtained from two-pool BMC solution with fixed $R_{2,SEI}$=40kHz.

We assumed Lorentzian line shapes for both pools, tests using a Gaussian line-shape for the SEI pool led to similar results and no improvement of $R_{2,SEI}$ (data not shown).

An additional deviation in the quantification could originate from bulk metal environments that contribute to the NMR spectrum but are not taking part in the exchange process (see discussion above when discussing non-surface Li). To test whether that affects the conclusions, the BMC fits were performed for selected datasets (two temperatures for LP30 and LiTFSI/DME/DOL systems), assuming an additional exchange-inactive Li dendrites fraction of 20%. The induced deviations in the fitting results were of similar order of magnitude and direction as the inactive metal fraction (i.e. approximately 20% higher). Nevertheless, the relative trends between

datasets, such as increasing exchange rate with temperature or relative differences between the two electrolyte systems remained.

6. Variable temperature Z-spectra for LP30

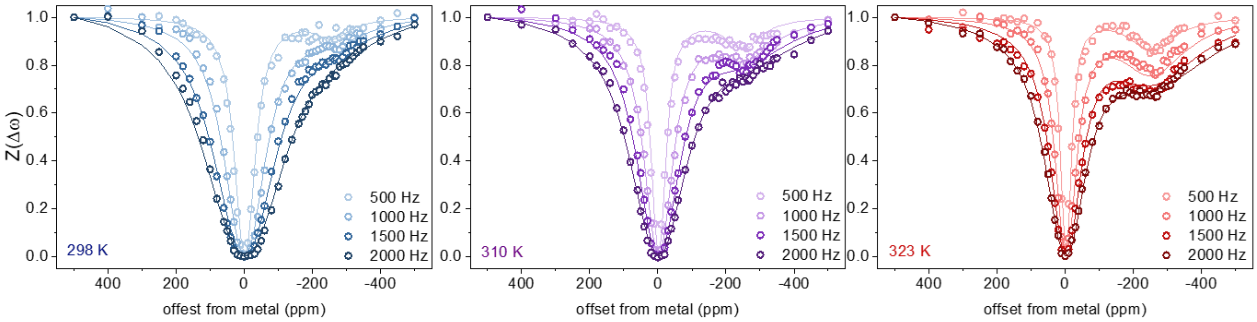

Figure S9 Z-spectra obtained for dendrites in LP30 with 0.2 sec saturation pulse with varying power level at 298, 310 and 323 K.

In order to determine the activation energy for Li migration across the metal SEI interface, Z-spectra were measured for the LP30 system on one sample at three different temperatures – 298, 310 and 323 K (Figure S9). The exchange rates were determined based on the analytical model and the activation barrier, E_a, was determined from the slope of a linear fit (Figure S10):

$$\ln(k_{metal-SEI}) = -\left(\frac{E_a}{k_B}\right)\left(\frac{1}{T}\right) - \ln{(A)}$$

With $k_B$the Boltzmann constant, T, the temperature and A the Arrhenius pre-exponential factor.

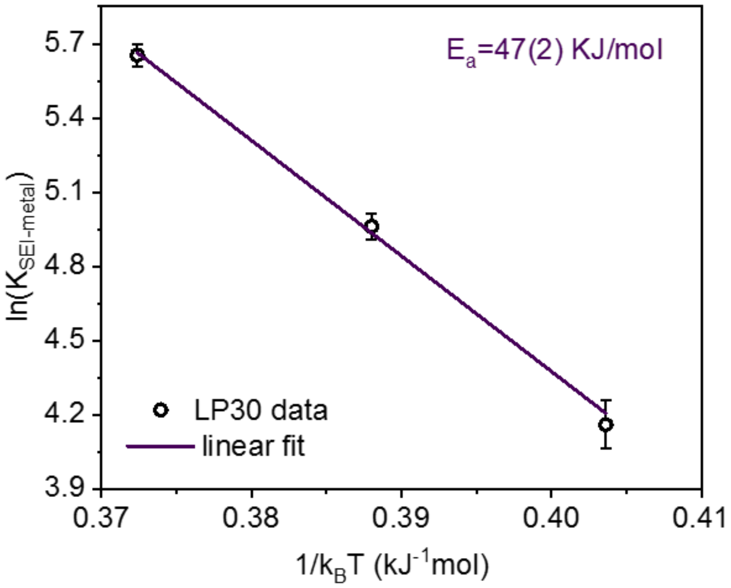

Figure S10 Determination of the activation barrier for lithium migration across the SEI-metal interface extracted based on the Arrhenius equation.

7. Electrochemistry data

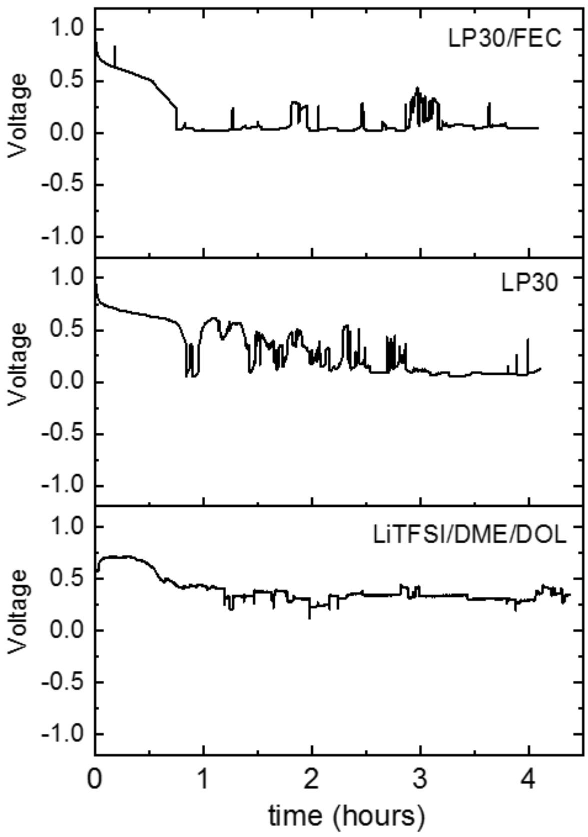

Figure S11 Voltage profiles of galvanostatic discharge of 4 hours with 0.5mA of symmetric lithium electrodes in the electrochemical set up in the NMR tube in different

For CEST experiments dendrites were grown in the NMR tube using the set up described above and shown in Figure S4. A constant current of 0.5mA was used in all experiments (corresponding to about 3.3 mA/cm^2) and applied for 4 hours. Voltage profiles measured in the different electrolytes corresponding to the systems used in the variable temperature CEST experiments are shown in Figure S11. We note that the set up was optimized for forming dendrites in the NMR tube and is not optimal for studying the electrochemical performance in different electrolytes. This is due to the confined space in the NMR tube and the separation of the two lithium electrodes by a Teflon insulator. This results in the observed voltage profiles that are strongly affected by transient short circuits formed due to movement of the dendrites within the liquid cell. In this case, we confirm and quantify the formation of dendritic lithium in the three systems through the unique properties of the dendrites compared to bulk lithium in NMR measurements (Figure 2 in the main text). Comparison of the electrochemical performance in LP30 and LP30/FEC electrolytes is based on lithium plating on Cu done in standard coin cells (Figure S12).

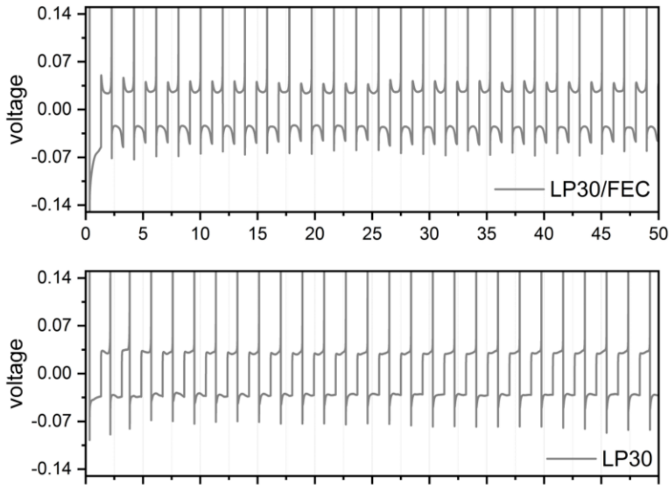

Figure S12 Galvanostatic lithium plating and stripping on a Cu foil cycled vs. lithium metal with 0.5mA/cm^2 in LP30 and LP30/FEC. The columbic efficiency obtained after 25 cycles was 90 and 95% for the two electrolytes respectively.

References

(1) Fairhurst, D.; Cosgrove, T.; Prescott, S. W. Relaxation NMR as a Tool to Study the Dispersion and Formulation Behavior of Nanostructured Carbon Materials. *Magn. Reson. Chem.* **2016**, *54* (6), 521–526. https://doi.org/10.1002/mrc.4218.

(2) Weber, R.; Cheng, J.-H.; Louli, A. J.; Coon, M.; Hy, S.; Dahn, J. R. Surface Area of Lithium-Metal Electrodes Measured by Argon Adsorption. *J. Electrochem. Soc.* **2019**, *166* (14), A3250–A3253. https://doi.org/10.1149/2.1181913jes.

(3) McConnell, H. M. Reaction Rates by Nuclear Magnetic Resonance. *J. Chem. Phys.* **1958**, *28* (3), 430–431. https://doi.org/10.1063/1.1744152.

(4) Woessner, D. E.; Zhang, S.; Merritt, M. E.; Sherry, A. D. Numerical Solution of the Bloch Equations Provides Insights into the Optimum Design of PARACEST Agents for MRI. *Magn. Reson. Med.* **2005**, *53* (4), 790–799. https://doi.org/10.1002/mrm.20408.

(5) Zaiss, M.; Bachert, P. Exchange-Dependent Relaxation in the Rotating Frame for Slow and Intermediate Exchange - Modeling off-Resonant Spin-Lock and Chemical Exchange Saturation Transfer. *NMR Biomed.* **2013**, *26* (5), 507–518. https://doi.org/10.1002/nbm.2887.
